# Perineal schwannoma

**DOI:** 10.1186/s13104-016-2108-1

**Published:** 2016-06-13

**Authors:** Anass Majbar, Abdelmalek Hrora, Ahmed Jahid, Mohamed Ahallat, Mohamed Raiss

**Affiliations:** Faculty of Medicine and Pharmacy, Surgery, Mohamed V University, Rabat, Morocco; Surgery Unit C, Ibn Sina University Hospital, Rabat, Morocco; Pathology Unit, Ibn Sina University Hospital, Rabat, Morocco

**Keywords:** Perineum, Schwannoma, Anal sphincter

## Abstract

**Background:**

Schwannoma is a benign tumor arising from Schwann cells of the peripheral nerve sheath. Perineal schwannomas are exceptional, and rarely reported in the literature. We report a case of a perineal schwannoma, close to the anal sphincter, and provide a short summary of clinical, radiological and surgical features of this rare entity.

**Case presentation:**

A 62 year-old male patient was admitted for a suspected perineal mass. At clinical examination, he had a soft mass, located on the right of the anus. Computed tomography showed a perineal mass, located on the right side of the anal sphincter that enhanced after injection of the contrast medium. Complete surgical excision of the tumor was performed. The most challenging part during the surgery was the dissection and preservation of the anal sphincter to avoid anal incontinence. Pathologic examination revealed a completely excised schwannoma.

**Conclusions:**

Perineal schwannomas are very rare tumors that are usually asymptomatic, and which present as large masses. Complete excision is necessary to avoid recurrences. Surgical resection may be difficult depending of proximity to the anal sphincter. A cautious dissection in such cases is required in order to reduce the risk of incontinence.

## Background

Schwannomas are benign tumours arising from Schwann cells of the peripheral nerve sheath. They are most frequently present in young male patients aged 20–50 years [1]. Schwannomas are usually solitary slow growing, non-aggressive neoplasms, discovered as large masses [2]. They comprise 5 % of all benign soft tissue tumours and are usually located in the head and neck and flexor surfaces of the upper and lower extremities [2]. Perineal schwannomas are exceptional, and only few cases were reported in the literature [3].

We report a case of a perineal schwannoma in close proximity to the anal sphincter, and we provide a summary of clinical, radiological and surgical features of this rare entity.

## Case description

**Fig. 1:**
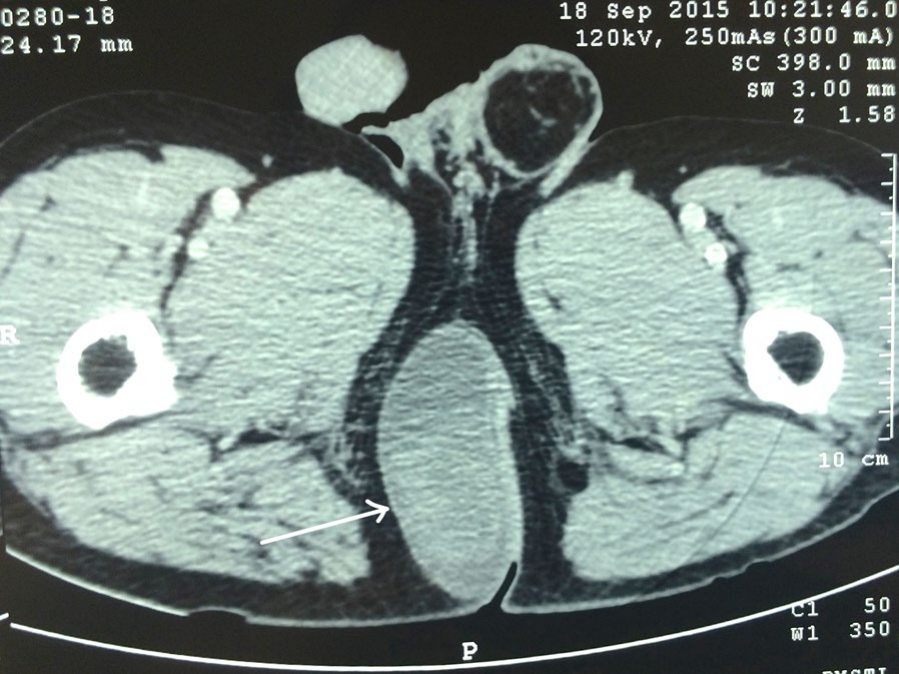
Computed tomography showing the perineal mass, partially enhanced after injection of contrast medium (*arrow*)

A 62 years-old male patient was admitted for a perineal mass. This mass appeared eight months before admission, and its volume had increased rapidly in the last few weeks. The mass was not painful, and there was no rectal bleeding or bowel disorders. Clinical examination showed a soft mass, located laterally to the right of the anus. Rectal examination showed no anomaly in the anal sphincter. Ultrasonography revealed a well-defined heterogeneous tumor, with increased vascularity and partially cystic. Computed tomography (Fig. 1) showed a well-defined perineal mass, located on the right side of the anal sphincter with the presence of cystic areas. The mass enhanced after injection of the contrast medium.

**Fig. 2:**
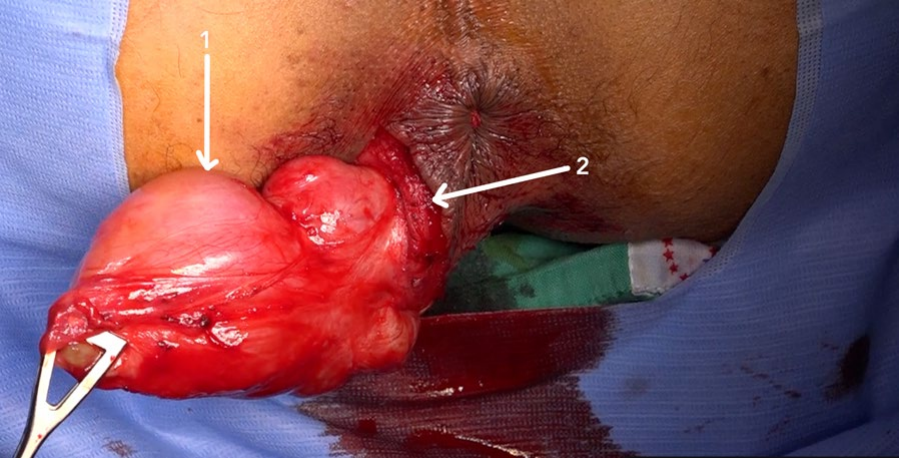
Surgical view showing the perineal mass close to the external anal sphincter (1: the perineal tumour, 2: anal sphincter fibers)

The patient underwent a complete excision of the tumor. The patient was placed in a lithotomy position, and surgery started with an arciform incision on the right perianal area. Surgical exploration showed a white-yellowish encapsulated tumor with elastic consistency, measuring 11 × 7 cm. The mass was dissected from surrounding tissues, preserving the muscular fibers of the anal sphincters (Fig. 2). The postoperative course was uneventful.

**Fig. 3:**
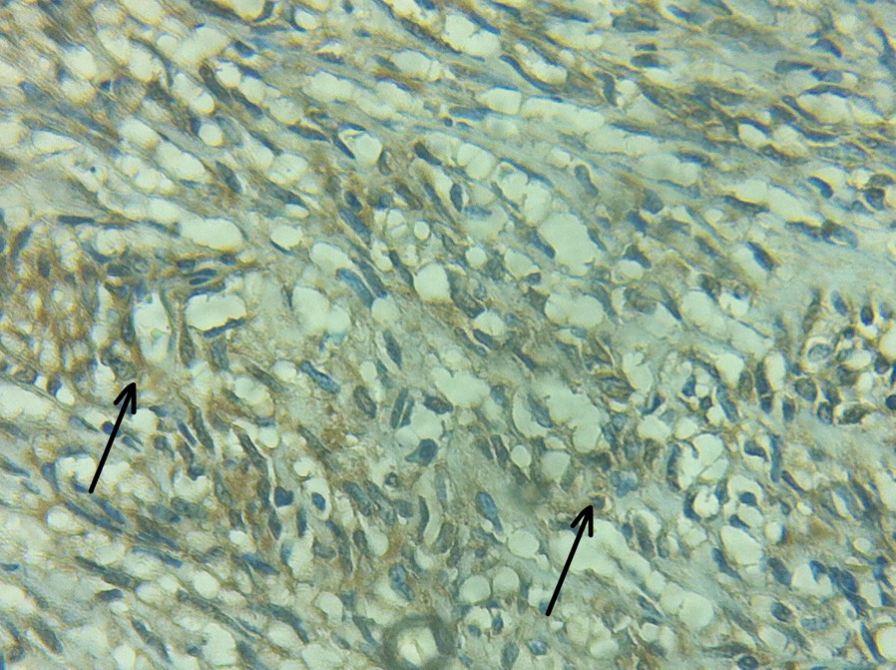
Tumor cells demonstrating nuclear and cytoplasmic immunoreactivity for S-100 (×20) (*arrows*)

Histopathological report revealed a proliferation of spindle cells having elongated or ovoid nuclei. On Immunohistochemical study (Fig. 3), tumor cells were reactive to S-100 antibody and negative for others antibodies (CD 117; CD 34; SMA). The definitive diagnosis was a benign perineal schwannoma. No recurrence occurred after 6 months of follow-up.

## Discussion

Perineal schwannoma is a very rare tumor, with only few cases reported in the literature. The rate of reported pelvic schwanommas is 1 % [4]. There are mostly benign and have a very low rate of malignant transformation [5].

Schwannomas are typically slow growing and non-aggressive. Therefore, they are usually asymptomatic and discovered as large perineal masses [6, 7]. When symptomatic, patients may present with non-specific pain, palpable mass or rectal dysfunction [8]. Malignant schwannomas are usually large, infiltrating and fast-growing tumors [9].

Preoperative diagnosis is particularly challenging. On computed tomography, schwannomas usually have an ovoid or spherical shape with a smooth, well-defined border [2]. In up to 61 % of cases, they can present cystic changes as shown in the presented case. Schwannomas has a smooth and regular border, which allows differentiating them from other malignant masses such as sarcomas. When schwannomas are mostly solid, the differential diagnosis includes neurofibroma and lymphoma [2]. The MRI characteristics of peripheral schwannomas typically include hypointensity on T1-weighted images and hyperintensity on T2-weighted images [2, 10]. MRI is also useful for analysing the tumor’s relationships with adjacent structures in the perineum.

Perineal schwannomas should be excised completely [5, 11]. The most challenging part in the presented case was the proximity of the anal sphincter. A cautious dissection in such cases is mandatory to avoid an injury of the muscular fibers of the sphincter and expose the patient to the risk of incontinence. Some authors advised a partial resection when the tumor is in proximity to others organs to avoid iatrogenic damage. However, recurrences are related to incomplete resections.

The final diagnosis is made by histopathology. Benign schwannomas consist of compact cellular lesions with interlacing and cellular fascicles (Antoni A) and less cellular and myxoid areas (Antoni B) [8]. Immunohistochemical analysis is very useful to differentiate schwannomas from other perineal masses, by showing a positive and uniform S-100 staining [4, 8]. Malignant schwannomas are characterized histologically by perineural and intraneural spreads, herniation into the lumina of the vessels and nuclear palisading [2].

## Conclusions

In summary, perineal schwannomas are very rare tumors, usually asymptomatic, presenting as large masses. Surgical resection may be difficult in the case of close proximity to the anal sphincter. A cautious dissection in such cases is necessary to reduce the risk of incontinence.

## Abbreviations

CT: computed tomography
MRI: magnetic resonance imaging
